# Hypermethylation of *UCHL1* Promotes Metastasis of Nasopharyngeal Carcinoma by Suppressing Degradation of Cortactin (CTTN)

**DOI:** 10.3390/cells9030559

**Published:** 2020-02-27

**Authors:** Yin Zhao, Yuan Lei, Shi-Wei He, Ying-Qin Li, Ya-Qin Wang, Xiao-Hong Hong, Ye-Lin Liang, Jun-Yan Li, Yang Chen, Wei-Jie Luo, Pan-Pan Zhang, Xiao-Jing Yang, Qing-Mei He, Jun Ma, Na Liu, Ling-Long Tang

**Affiliations:** Sun Yat-sen University Cancer Center, State Key Laboratory of Oncology in South China, Collaborative Innovation Center of Cancer Medicine, Guangdong Key Laboratory of Nasopharyngeal Carcinoma Diagnosis and Therapy, Guangzhou 510060, China; zhaoyin@sysucc.org.cn (Y.Z.); leiyuan@sysucc.org.cn (Y.L.); hesw@sysucc.org.cn (S.-W.H.); liyingq@sysucc.org.cn (Y.-Q.L.); wangyaq@sysucc.org.cn (Y.-Q.W.); hongxh@sysucc.org.cn (X.-H.H.); liangyl@sysucc.org.cn (Y.-L.L.); lijy1@sysucc.org.cn (J.-Y.L.); chenyang1@sysucc.org.cn (Y.C.); luowj1@sysucc.org.cn (W.-J.L.); zhangpp@sysucc.org.cn (P.-P.Z.); yangxiaoj@sysucc.org.cn (X.-J.Y.); heqm@sysucc.org.cn (Q.-M.H.); majun2@mail.sysu.edu.cn (J.M.); liun1@sysucc.org.cn (N.L.)

**Keywords:** nasopharyngeal carcinoma, UCHL1, metastasis, methylation, CTTN

## Abstract

Epigenetic regulation plays an important role in the development and progression of nasopharyngeal carcinoma (NPC), but the epigenetic mechanisms underlying NPC metastasis remain poorly understood. Here, we demonstrate that hypermethylation of the *UCHL1* promoter leads to its downregulation in NPC. Restoration of UCHL1 inhibited the migration and invasion of NPC cells in vitro and in vivo, and knockdown of UCHL1 promoted NPC cell migration and invasion in vitro and in vivo. Importantly, we found that UCHL1 interacts with CTTN, and may function as a ligase promoting CTTN degradation by increasing K48-linked ubiquitination of CTTN. Additionally, restoration of CTTN in NPC cells that overexpressed UCHL1 rescued UCHL1 suppressive effects on NPC cell migration and invasion, which indicated that CTTN is a functional target of UCHL1 in NPC. Our findings revealed that UCHL1 acts as a tumor suppressor gene in NPC and thus provided a novel therapeutic target for NPC treatment.

## 1. Introduction

Nasopharyngeal carcinoma (NPC) is a malignant epithelial tumor that arises from the nasopharynx. According to global cancer statistics, there were 129,079 new cases of NPC worldwide in 2018, and 72,987 people died of this cancer [1]. The onset of intensity modulated radiotherapy and combined chemoradiotherapy has resulted in improved prognosis for patients with NPC, and the main cause of treatment failure of NPC is distant metastasis [2]. Thus, efforts to better understanding the molecular mechanisms underlying NPC metastasis will help to get novel therapeutic strategies to improve treatment of metastatic NPC.

Ubiquitin C-terminal hydrolase L1 (UCHL1) belongs to ubiquitin C-terminal hydrolase (UCH) subclass of deubiquitinating enzymes (DUBs) [3]. UCHL1 has both hydrolase activity and ligase activity [4]. UCHL1 is first reported to play an important role in the pathogenesis of Alzheimer’s disease [5]. UCHL1 has also been reported to act as either an oncogene or tumor suppressor gene in different types of cancer [6,7,8,9]. Hussain et al. report that UCHL1 promotes MYC-induced lymphomas by promoting the assembly of eIF4F and stimulating protein synthesis [9] UCHL1 is also highly expressed in lung cancer, and can promote metastasis by enhancing HIF-1α stability by deubiquitylating HIF-1α [6]. Hypermethylation of the UCHL1 promoter leads to low UCHL1 expression, and promotes cell proliferation by regulating the stability of p53 and MDM2 in hepatocellular cancer (HCC) and NPC [7,8]. Nevertheless, the specific role of UCHL1 in NPC metastasis, in particular which enzyme activity of UCHL1 is important in NPC metastasis, has not been investigated yet.

Cortactin (CTTN) is a ubiquitous actin-binding protein that was originally identified as a substrate of the Src kinase [10,11]. CTTN is over-expressed in many types of cancer, and upregulation of CTTN contributes to the tumorigenesis of many malignancies, including HCC [12,13], head and neck squamous cell carcinomas, breast cancer [14], and esophageal squamous cell carcinoma [15]. Not surprisingly, dysregulation of CTTN expression caused by post-translation modification can contribute to the pathogenesis of human diseases, including cancer. For example, HDAC6-mediated deacetylation of CTTN is important for the alleviation of thrombocytopenia [16]. Moreover, Src-mediated phosphorylation of CTTN can promote tumor cell migration in HCC [13]. However, the mechanism of ubiquitin regulation of CTTN is far from clear.

In this study, UCHL1 promoter hypermethylation was confirmed in NPC tissues by bisulfite pyrosequencing, corroborating our previous work and public microarray data. Moreover, UCHL1 was significantly downregulated in NPC exhibiting promoter hypermethylation. Restoration of UCHL1 suppressed NPC invasion and metastasis in vitro and in vivo. Further investigation indicated that UCHL1 interacts with CTTN, functioning as a ubiquitin ligase and mediating CTTN degradation by increasing K48-linked ubiquitination of CTTN. Therefore, this study confirms that UCHL1 occupies a tumor suppressor role in NPC metastasis by mediating CTTN degradation, and may represent a novel therapeutic target for NPC treatment.

## 2. Materials and Methods

### 2.1. Clinical Specimens and Cell Culture

Twenty-seven freshly-frozen NPC biopsy tissue samples and twenty-five normal nasopharynx tissue samples were obtained from the Sun Yat-sen University Cancer Center (Guangzhou, China). This study was approved by the Institutional Ethical Review Boards of the Sun Yat-sen University Caner Center, and every patient provided informed consent. Six NPC cell lines (CNE2, SUNE1, HONE1, HNE1, 5-8F, and 6-10B) were cultured in RPMI-1640 (Invitrogen, Carlsbad, CA, USA) supplemented with 10% FBS (ExCell Bio, Shanghai, China), and two normal nasopharyngeal epithelial cell lines (NP69 and N2-Tert) were grown in KSFM (Invitrogen) supplemented with bovine pituitary extract (BD Biosciences, Franklin Lake, NJ, USA). NP69, N2-Tert and all NPC cell lines had been authenticated and were gifts from Dr. Mu-sheng Zeng (Sun Yat-sen University Cancer Center, China) [17,18]. HEK293T cells were grown in DMEM supplemented with 10% FBS, and were obtained from ATCC. All the cells were authenticated using shot-tandem repeat profiling, tested for *Mycoplasma* contamination, and cultured for less than 2 months.

### 2.2. Antibodies and Reagents

Anti-CTTN (Proteintech, Whuhan, China, 11381-1-AP), HRP-conjugated goat anti-mouse IgG (CST, Boston, MA, USA, 7076), HRP-conjugated goat anti-rabbit IgG (CST, 7074), anti-GFP (Proteintech, 50430-2-AP), anti-α-tubulin (Proteintech, 11224-1-AP), anti-FLAG (Sigma, St. Louis, MO, USA, F3165), anti-HA (Proteintech, 51064-2-AP), anti-UCHL1 (CST, 13179S), anti-Ub (Santa Cruz, Dallas, TX, USA, sc-8017), Goat-anti-mouse Alexa Fluor ®488 IgG (Life Technologies, Carlsbad, CA, USA, A-10684), Goat-anti-rabbit, and Alexa Fluor ®594 IgG (Life Technologies, A-11079), MG132 (Sigma, 474787), DAC (Sigma, 189825), CHX (Sigma, 5087390001), and puromycin (Thermo, Waltham, MA, USA, A1113802) were purchased from the indicated manufacturers.

### 2.3. DNA Extraction and Bisulfite Pyrosequencing

Genomic DNA was extracted using an AllPrep RNA/DNA Mini kit (Qiagen, Dusseldorf, Germany) following manufacturer’s instructions. Bisulfite transformation and purification of 1-2 μg DNA were performed with an EpiTect Bisulfite Kit (Qiagen) according to the manufacturer’s instructions. The primers for PCR amplification and bisulfite pyrosequencing were designed by the PyroMark Assay Design Software 2.0 (Qiagen), and listed in Table S1. PCR reactions were incubated at 95 °C for 3 min, followed by 50 cycles of 95 °C for 15 s, 54 °C for 20 s and 72 °C for 30 s, and then a final hold at 72 °C for 5 min. The pyrophosphate sequencing reactions were conducted on a PyroMark Q96 ID system (Qiagen).

### 2.4. RNA Extraction and Quantitative RT-PCR

Cells or tissues were collected in TRIzol reagent (Invitrogen) for RNA extraction. First-strand cDNA synthesis was performed using a reverse-transcription kit (Promega, Madison, Wis., USA) and random primers (Promega). Quantitative PCR reactions were conducted on an SFX (96) system (Bio-Rad, Hercules, CA, USA) with 2×SYBR Green mix (Life Technologies). The sequences of primers are listed in Supplementary Table S1. Data was normalized to the expression of the GAPDH gene.

### 2.5. Immunoblotting

Cells were lysed in NP-40 lysis buffer supplied with protease and phosphatase inhibitor cocktail (Roche, Basle, Switzerland) on ice for 5 min. The lysates were centrifuged at 12000 rpm for 5 min at 4 °C. The protein supernatants were mixed with SDS loading buffer were heated at 95 °C for 10 min and then separated with SDS-PAGE. The proteins were electrophoretically transferred to polyvinylidene fluoride (PVDF) membranes (Millipore, Burlington, MA, USA). The membranes were blocked with 5% wt/vol skim milk. Primary antibodies and second antibodies were diluted in corresponding antibody diluent (Beyotime, Shanghai, China), and membranes were incubated with the appropriate antibodies prior to immunoblot analysis.

### 2.6. Transient Transfection

Phage-puro-6tag-UCHL1, phage-puro-6tag-UCHL1(C90S), phage-puro-6tag-CTTN, and PRK-GFP-CTTN were constructed by Y.Z. following molecular cloning guidelines. PRK-HA-Ub, PRK-HA-Ub(K48O), and PRK-HA-Ub(K63O) were generously provided by Professor Bo Zhong (Wuhan University, Whuhan, China). Plasmid transfection was carried out using Lipofectamine 3000 (Invitrogen) following the manufacturer’s instructions. Cells were transfected for 24–48 h prior to being used in experiments.

### 2.7. Lentivirus-Mediated Gene Transfer

HEK293 cells were transfected with pLKO.1-shUCHL1, phage-6tag-UCHL1, or the empty vector along with the packaging vectors pSPAX2 and pMD2G. Culture medium was changed with fresh full medium after 8 hours. After 48 h, the supernatants were harvested and used to infect SUNE1 or HONE1 cell lines followed by puromycin selection for one week. The infection efficiency was confirmed by quantitative RT-PCR and immunoblotting.

### 2.8. Wound Healing Assay

Cells were seeded at confluent density in 6-well plates and culture medium was replaced with serum-free medium. After cells were starved for 24 h, 200 μL tips were used to create linear wounds in the cell monolayers, and cells were incubated for another 24 h in starvation medium. Images of the wounded areas were captured by a microscope (Leica, Wetzlar, Germany) with open field at 0 h and 24 h for further analysis.

### 2.9. Migration and Invasion Assays

Cells (5 × 10^4^ or 1 × 10^5^) suspended in 200 μL of serum-free medium were seeded into transwell chambers (8 μm pores, Corning, NY, USA) that were either pre-coated with Matrigel (BD Biosciences) (invasion assay) or that lacked Matrigel (BD Biosciences) (migration assay). The chambers were placed into 24-well plates with full media containing 10% FBS and cultured at 37 °C. After 12 h (migration assay) or 24 h (invasion assay), cells were fixed with methyl alcohol and stained with crystal violet. Random visual fields (n = 10) of each well were obtained by a microscope (Leica) with open field for further analysis.

### 2.10. Mass Spectrometry and Co-Immunoprecipitation (co-IP)

Cells were lysed and protein lysates were incubated with anti-IgG or anti-FLAG antibodies overnight at 4 °C. The immune complex was recovered using protein A/G Sepharose beads (Santa Cruz). After washing and denaturation, the immune complex was separated using SDS-PAGE, and the indicated bands were subjected to mass spectrometry analysis. For co-IP, the lysates were immunoprecipitated with anti-IgG or the indicated antibodies overnight at 4 °C. The precipitant was recovered by magnetic beads (Invitrogen) for 1 h at room temperature, and washed three times with wash buffer followed by immunoblotting analysis.

### 2.11. Immunofluorescence Staining

SUNE1 cells were seeded in a 24-well plate coated with Teflon glass-climbing. After 24 h, cells were washed, fixed, and permeabilized with 0.5% Triton X-100. Subsequently, cells were blocked with PBS containing 1% FBS, incubated with the indicated primary antibodies, and then stained with fluorescent-combined secondary antibodies. 4’,6-diamidino-2-phenylindole (DAPI; Sigma) was used to stain nuclei, and a FV1000 microscope (Olympus, Tokyo, Japan) was used to obtain the fluorescence images.

### 2.12. Popliteal Lymph Node Metastasis Models

5-week-old female BALB/c nude mice were obtained from the Medical Experimental Animal Center of Guangdong Province (Guangzhou, China). 3×10^5^ SUNE1 cells stably transfected with plasmids encoding shControl or shUCHL1 were injected into the footpads of mice on day 0. After 35 days, the mice were sacrificed, and then the primary footpad tumors and popliteal lymph nodes were collected and fixed in formalin solution. Sections of the primary tumors and lymph nodes were then processed for H&E staining. Metastatic tumor cells in the lymph nodes were stained with anti-pan-cytokeratin antibody. Images were captured by an AxioVision Rel.4.6 computerized image analysis system (Carl Zeiss, Jena, Germany).

### 2.13. In Vivo Xenograft Tumor Models

5-week-old female BALB/c nude mice were obtained from the Medical Experimental Animal Center of Guangdong Province (Guangzhou, China). 1 × 10^6^ SUNE1 cells stably overexpressing UCHL1 or control vector were injected into the mice by the tail vein. The survival of the mice was monitored every day. After 8 weeks, the mice were euthanized and lung tissues were collected. The lung tissues were fixed in formalin solution and processed for H&E staining. The stained sections were analyzed with a Leica O8 microscope under a 20× objective. The intensities of staining were quantified and analyzed using the ViewPointBETA v1 software.

### 2.14. Immunohistochemistry Assay

Formalin-fixed paraffin-embedded (FFPE) slices of xenograft mice tissues were used to perform Immunohistochemistry (IHC). Briefly, the tissues were deparaffinized at 60 °C for 30 min and then rehydrated. The endogenous peroxidase was blocked by 3% H2O2, and the tissues were treated by high-temperature citrate for antigen retrieval. Subsequently, non-specific binding was blocked and tissues were incubated with primary antibodies at 4 °C overnight.

### 2.15. Ethics Statement in Animal Study

This study has been approved by the Institutional Ethical Review Boards of the Sun Yat-sen University Cancer Center (Ethical code number: GZR2019-022), and the written informed consents have been obtained from all patients. All animal studies have been approved by the Institutional Animal Care and Use Ethics Committee of Sun Yat-sen University Cancer Center (Ethical code number L102042018110I).

### 2.16. Statistical Analysis

Differences between groups were tested using unpaired two-tailed Student’s *t*-test and one-way ANOVA or two-way ANOVA analysis with Prism 5 (GraphPad Inc., San Diego, CA, USA). All data are presented as the mean ±S.D. were extracted from no less than three independent experiments. A p value of less than 0.05 was considered statistically significant. For animal survival analysis, the Kaplan-Meier method was used to generate graphs and the survival curves were analyzed by log-rank analysis.

## 3. Results

### 3.1. The UCHL1 Promoter is Hypermethylated in NPC

**Figure 1:**
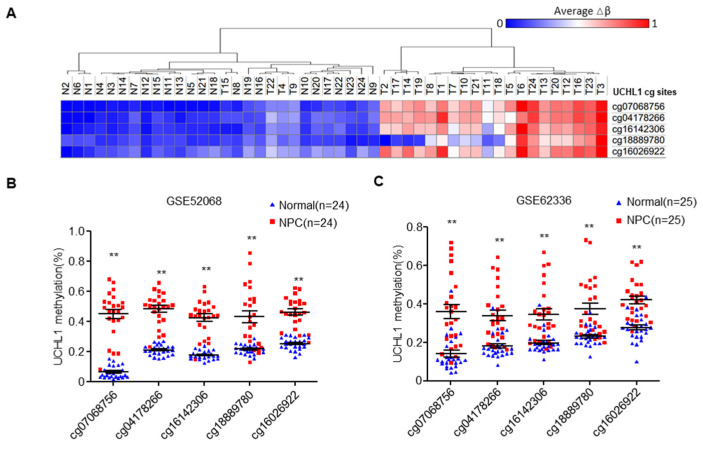

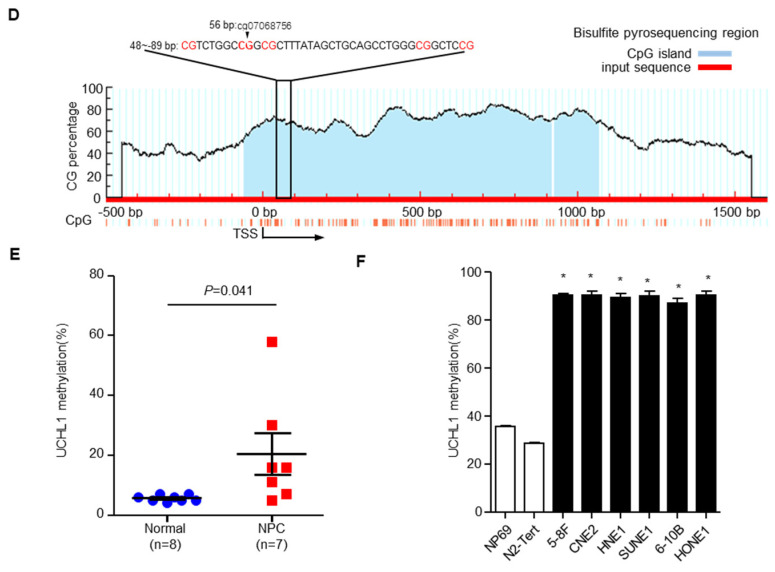
The UCHL1 is hypermethylated in nasopharyngeal carcinoma. (**A**) Heatmap cluster of UCHL1 CG sites between NPC (n = 24) and normal nasopharyngeal tissue samples (n = 24). (**B**,**C**) The methylation level of UCHL1 cg sites in the GSE52068 (B) and GSE62336 (C) microarray data between NPC and normal nasopharyngeal tissue samples. (**D**) Schemtic of CpG islands and bisulfite pyrosequencing region in the UCHL1 promoter. Red region, input sequence; Blue region, CpG islands; TSS, transcription start site; cg07068756: the cg sites of UCHL1 identified in our previous genome-wide methylation microarray; red text: CG sites for bisulfite pyrosequencing; bold red text, the most methylated CG sites in UCHL1. (**E**,**F**) The methylation levels of the UCHL1 promoter region as determined by bisulfite pyrosequencing analysis in normal (n = 8) and NPC (n = 7) tissues (E), in NPEC (NP69, N2-Tert) and NPC (5-8F, CNE2, HNE1, SUNE1, 6-10B, HONE1) cell lines (F). * *p* < 0.05, ** *p* < 0.01; Mean ± S.D.; Student’s *t*-test.

Based on our previous genome-wide DNA methylation microarray data (GSE52068), we analyzed the methylation status of UCHL1, and investigated the frequency of hypermethylation of UCHL1 in NPC tissues (Figure 1A). We identified five significantly hypermethylated CpG sites in UCHL1, which were confirmed by another microarray analysis from Hong Kong (GSE62336, Figure 1B,C). To validate the dysregulated methylation status of UCHL1, we performed bisulfite pyrosequencing analysis of cg07068756 (the most significantly hypermethylated CpG site ranked by p value; CpG islands and the bisulfite pyrosequencing region of UCHL1 are shown in Figure 1D) in normal and NPC tissue. The methylation level of UCHL1 was significantly higher in NPC tissue compared with normal tissue (Figure 1E and Supplementary Figure S1). Consistently, UCHL1 methylation was elevated in NPC cell lines (Figure 1F and Supplementary Figure S2). Collectively, these data suggest that UCHL1 is hypermethylated in NPC.

### 3.2. UCHL1 Hypermethylation Contributes to Downregulation of UCHL1 Expression

**Figure 2:**
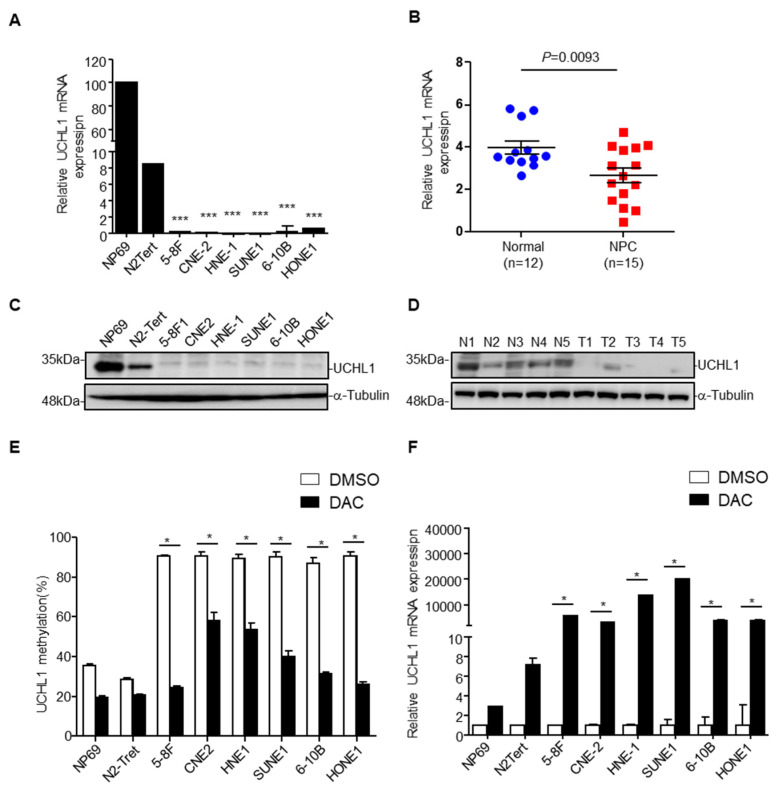
Promoter hypermethylation mediates downregulation of UCHL1 in nasopharyngeal carcinoma. (**A**,**B**) qRT-PCR analysis of UCHL1 mRNA expression in normal nasopharyngeal epithelial cell lines (NP69 and N2-Tert) and nasopharyngeal carcinoma cell lines (5-8F, CNE2, HNE1, SUNE1, 6-10B, HONE1) (A), in normal (n = 12) and NPC (n = 15) tissues (B). (**C**,**D**) Immunoblot analysis of UCHL1 and α-tubulin protein expression in NP69 and nasopharyngeal carcinoma cell lines (C), in normal (N, n = 5) and NPC (T, n = 5) tissues (D). (**E**) UCHL1 methylation levels determined by bisulfite pyrosequencing analysis in NP69 and nasopharyngeal carcinoma cell lines treated with or without DAC. (**F**) Quantitative RT-PCR analysis of UCHL1 mRNA expression in NP69 and nasopharyngeal carcinoma cell lines treated with or without DAC. * *p* < 0.05; *** *p* < 0.001; Mean ± S.D.; Student’s *t*-test. Data are representative of at least three independent experiments.

To investigate the causal link between UCHL1 promoter methylation and expression, we examined UCHL1 expression by qRT-PCR and immunoblotting assays in 6 NPC and 2 normal nasopharyngeal epithelial cell lines, and in 20 NPC and 17 normal nasopharynx tissue samples. UCHL1 mRNA was significantly decreased in NPC cell lines and tissues (Figure 2A,B). Moreover, analysis of the TCGA database showed that UCHL1 mRNA was reduced in several types of cancer, including colon adenocarcinoma, kidney renal clear cell carcinoma, kidney renal papillary cell carcinoma, and bladder urothelial carcinoma (Supplementary Figure S3A). In addition, immunoblot analysis showed that UCHL1 protein levels were also significantly lower in NPC cell lines and tissues (Figure 2C,D). Taken together, these results indicate that UCHL1 is downregulated in NPC and other tumor types.

Next, we investigated whether the downregulation of UCHL1 was caused by its promoter hypermethylation. After treatment with the demethylating drug DAC (inhibitor of DNA methyltransferase), UCHL1 methylation levels were decreased in the NPC cell lines (Figure 2E and Supplementary Figure S2). Conversely, the mRNA levels of UCHL1 were substantially increased (Figure 2F). MethHC database analysis found that UCHL1 methylation level was significantly increased in colon adenocarcinoma, kidney renal clear cell carcinoma, kidney renal papillary cell carcinoma, and bladder urothelial carcinoma Supplementary Figure S3B,C), negatively correlating with UCHL1 mRNA expression. These findings illustrate that UCHL1 downregulation results from its promoter hypermethylation in NPC and in other tumor types.

### 3.3. UCHL1 Suppresses NPC Cell Migration and Invasion In Vitro

**Figure 3:**
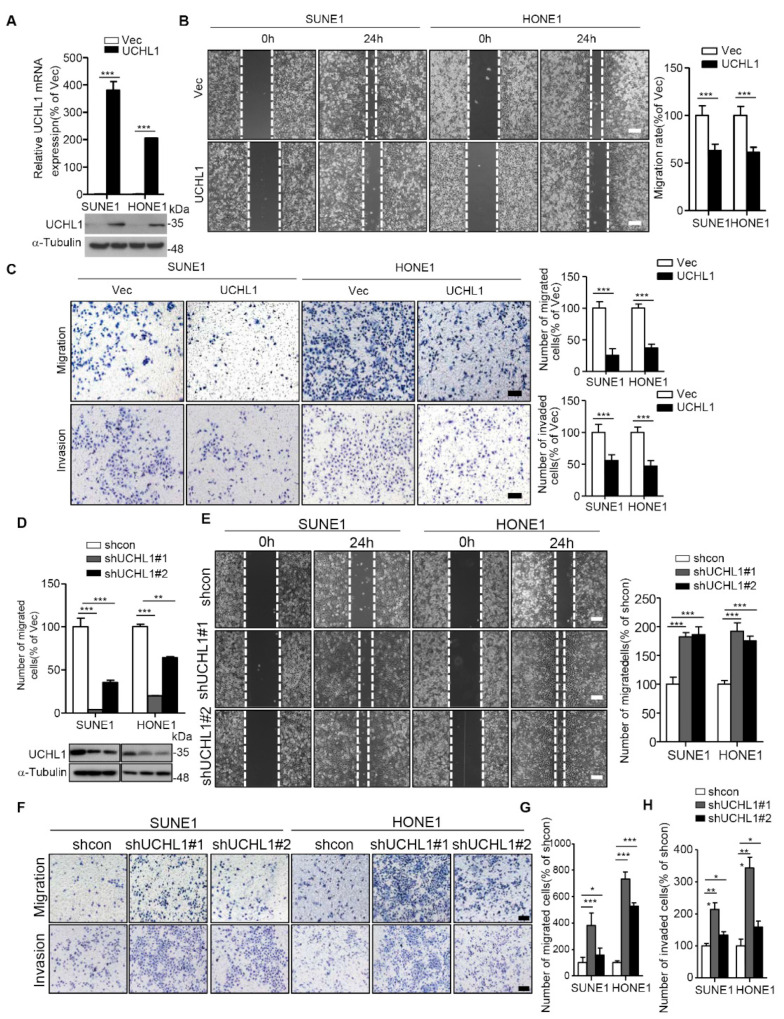
UCHL1 inhibits the migration and invasion of NPC cells in vitro. (**A**) qRT-PCR analysis of UCHL1 mRNA (up) and immunoblot analysis of UCHL1 and α-tubulin (down) in SUNE1 and HONE1 cells transfected with empty vector or plasmid encoding UCHL1. (**B**,**C**) Cell migration was measured using a wound healing assay (×200) (**B**) and transwell assay (×200) without Matrigel (**C**). Invasion was measured using a transwell assay with Matrigel (×200) (**C**). (**D**) qRT-PCR analysis of UCHL1 mRNA (up) and immunoblot analysis of UCHL1 and α-tubulin (down) in SUNE1 and HONE1 cells transfected with control or shUCHL1(#1 and #2) that stably overexpressed UCHL1. (**E**–**H**) Cell migration was measured using a wound healing assay (**E**) and transwell assay without Matrigel (**F**,**G**). Invasion was measured using a transwell assay with Matrigel (**F**,**H**). Scale bar:100 µm; Mean ± S.D.; * *p* < 0.05, ** *p* < 0.01; *** *p* < 0.001; Student’s *t*-tests. Data are representative of at least three independent experiments.

It has been reported that UCHL1 can inhibit the proliferation of NPC cells in vitro [7], but whether UCHL1 regulates the proliferation of NPC cells in vivo or the metastasis of NPC cells remains unclear. To determine whether UCHL1 regulates the migration and invasion of NPC cells, wound healing, transwell migration, and transwell invasion assays were performed on SUNE1 and HONE1 NPC cell lines. The SUNE1 and HONE1 cell lines were stably transfected with plasmids encoding an empty vector or UCHL1, and shcontrol or shRNA targeting UCHL1. Overexpression of UCHL1 significantly suppressed SUNE1 and HONE1 migration and invasion (Figure 3A–C). In contrast, knockdown of UCHL1 markedly increased SUNE1 and HONE1 migration and invasion (Figure 3D–H). These data indicate that UCHL1 inhibits NPC cell migration and invasion in vitro.

### 3.4. UCHL1 Interacts with CTTN

**Figure 4:**
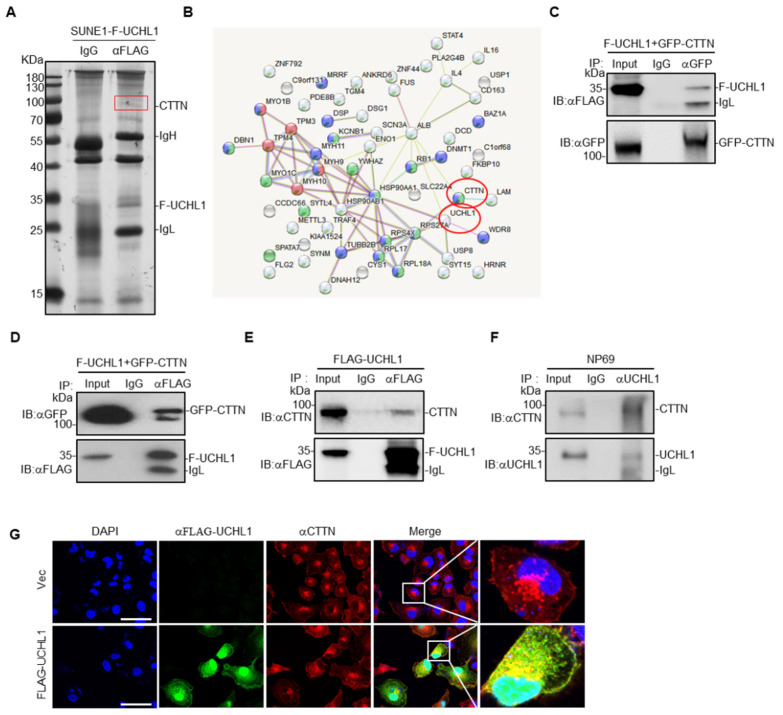
UCHL1 interacts with CTTN. (**A**)Immunoprecipitation (with anti-FLAG or IgG) and SDS-PAGE analysis of SUNE1 cells stably transfected with FLAG-UCHL1. (**B**) Mass spectrometry analysis of UCHL1 interacting proteins performed using the STRING website. (**C**) Immunoprecipitation (with anti-GFP or IgG) and immunoblot analysis (with anti-GFP and anti-FLAG) of SUNE1 cells transfected with plasmids encoding FLAG-UCHL1 and GFP-CTTN. (**D**) Immunoprecipitation (with anti-FLAG or IgG) and immunoblot analysis (with anti-GFP and anti-FLAG) of SUNE1 cells transfected with plasmids encoding FLAG-UCHL1 and GFP-CTTN. (**E**) Immunoprecipitation (with anti-FLAG or IgG) and immunoblot analysis (with anti-GFP and anti-CTTN) of SUNE1 cells transfected with plasmids encoding FLAG-UCHL1. (**F**) Immunoprecipitation (with anti-UCHL1 or IgG) and immunoblot analysis (with anti-UCHL1 and anti-CTTN) of NP69 cells. (**G**) Immunofluorescence analysis (with anti-FLAG, anti-CTTN or DAPI) of SUNE1 cells transfected with plasmids encoding FLAG-UCHL1. Scale bar: 50 μm.

To elucidate the mechanisms underlying the UCHL1-mediated suppressive effects in NPC, we co-immunoprecipitated FLAG-tagged UCHL1 in SUNE1 cells (Figure 4A), and then carried out mass spectrometry to identify its binding partners. CTTN was identified as a potential interacting partner of UCHL1 (Figure 4B and Supplementary Table S2). To further examine whether UCHL1 interacts with CTTN, we co-expressed FLAG-tagged UCHL1 with or without GFP-tagged CTTN in SUNE1 cells and performed co-immunoprecipitation experiments. We found that ectopic expressed FLAG-UCHL1 co-immunoprecipitated with ectopic expressed GFP-CTTN (Figure 4C,D), and ectopic expressed FLAG-UCHL1 could also readily pulled down endogenous CTTN (Figure 4E), which suggests that UCHL1 interacts with CTTN. To further validate the interaction between UCHL1 and CTTN, we performed endogenous immunoprecipitation experiments in NP69 cells which is of highly enriched of UCHL1 and found that two proteins at the endogenous levels bound to each other, which confirmed that UCHL1 and CTTN still have interaction (Figure 4F). To further substantiate this conclusion, we performed immunofluorescence staining, which showed co-localization of ectopic expressed UCHL1 and CTTN in the membrane and cytoplasm of SUNE1 cells (Figure 4G). Collectively, these data suggest that UCHL1 interacts with CTTN.

### 3.5. UCHL1 Targets CTTN for Ubiquitination and Degradation

**Figure 5:**
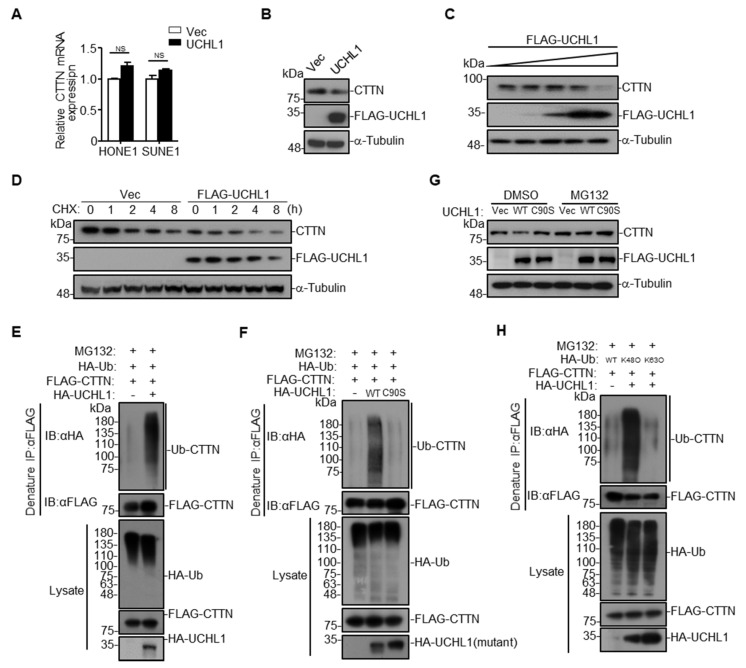
UCHL1 mediates CTTN degradation via K48-linked ubiquitination. (**A**) qRT-PCR analysis of CTTN mRNA expression of SUNE1 and HONE1 cells transfected with empty vector or plasmid encoding UCHL1. (**B**) Immunoblot analysis of CTTN, FLAG and a-tubulin of SUNE1 cells transfected with FLAG-UCHL1. (**C**) Immunoblot analysis (with anti-FLAG, CTTN, and α-Tubulin) of SUNE1 cells transfected with a dose of FLAG-UCHL1 (0 g, 0.5 μg, 1 μg, 2 µg, 4 μg). (**D**) Immunoblot analysis of FLAG, CTTN, and α-tubulin in SUNE1 cells transfected with empty vector, or FLAG-UCHL1(WT) for 24 h followed by treatment with CHX for 0–8 h. (**E**,**F**) Denature-immunoprecipitation (Denature-IP) (with anti-FLAG) and immunoblot analysis (with anti-FLAG, anti-HA, or anti-UCHL1) of SUNE1 cells transfected with plasmids encoding FLAG-CTTN, HA-Ubiquitin and empty vector, UCHL1(E), or UCHL1(C90S)(F) for 24 h followed by treatment with MG132 for 6 h. (**G**) Immunoblot analysis of FLAG, CTTN, and α-tubulin in SUNE1 cells transfected with empty vector, FLAG-UCHL1(WT) or FLAG-UCHL1(C90S) for 24 h followed by treatment with DMSO or MG132 for 6 h. (**H**) Denature-immunoprecipitation (Denature-IP) (with anti-FLAG) and immunoblot analysis (with anti-FLAG, anti-HA or anti-UCHL1) of SUNE1 cells transfected with plasmids encoding FLAG-CTTN and HA-Ubiquitin, HA-Ubiquitin(K48O), or HA-Ubiquitin(K63O) and empty vector or UCHL1 for 24 h followed by treatment with MG132 for 6 h. Mean ± S.D.; NS: no significance compared with vector; Student’s *t*-tests. Data are representative of at least three independent experiments.

To explore whether the interaction between UCHL1 and CTTN influences the expression of CTTN, qRT-PCR and western blotting assay were performed. We found that overexpression of UCHL1 had no effect on CTTN mRNA levels, but decreased its protein levels (Figure 5A,B). Further evaluation demonstrated that the levels of CTTN were decreased by increasing UCHL1 expression in a dose-dependent manner (Figure 5C). In addition, overexpression of UCHL1 promoted degradation of CTTN in the presence of CHX (Figure 5D).

These data suggest that UCHL1 targets CTTN for degradation. Our previous data showed that hypermethylation of UCHL1 leaded to its low-expression of mRNA and protein levels in NPC (Figure 2). Consistent with the finding that UCHL1 could promote the degradation of CTTN, we hypothesize that hypermethylation of UCHL1 could suppress the degradation of CTTN. To validate the hypothesis, immunoblotting assays were conducted in NP69, SUNE1 and HONE1 cells treated with or without DAC (inhibitor of DNA methyltransferase). We found that UCHL1 expression greatly increased, while CTTN expression decreased after DAC treatment. Besides, the protein level of CTTN in SUNE1 and HONE1 cells was significantly higher than in NP69 cells (Supplementary Figure S5). Taken together, we confirmed that hypermethylation of UCHL1 suppressed CTTN protein degradation in NPC.

UCHL1 itself has been reported to possess two opposite enzyme activities, hydrolase activity and ligase activity [4]. We therefore examined the effects of UCHL1 on the ubiquitination of CTTN, and we found that overexpression of UCHL1 increased CTTN ubiquitination (Figure 5E and Supplementary Figure S4A), suggesting that interaction between UCHL1 and CTTN may depend on the ligase activity of UCHL1. It has been reported that the UCHL1 (C90S) mutant is an enzymatically inactive mutant with significantly decreased ligase activity [19,20,21]. We constructed the UCHL1 (C90S) mutant, and found that overexpression of UCHL1, but not UCHL1 (C90S), could significantly increase the ubiquitination of CTTN (Figure 5F and Supplementary Figure S4B). Moreover, overexpression of UCHL1 but not UCHL1 (C90S) promoted CTTN degradation, and degradation of CTTN was completely blocked by the proteasome inhibitor MG132 (Figure 5G). These results indicate that UCHL1 regulates CTTN protein degradation through the ubiquitin-proteasome pathway.

Since Lys48-linked (K48-linked) poly-ubiquitination on a target protein results in proteasome degradation [22,23], we determined whether UCHL1 mediates K48-linked poly-ubiquitination on CTTN. As expected, mutation of the Lys48 site on ubiquitin (K48O) significantly increased UCHL1-mediated CTTN ubiquitination, whereas the K63O ubiquitin mutation had no effect (Figure 5H and Supplementary Figure S4C). These data collectively suggest that UCHL1 increased K48-linked poly-ubiquitination of CTTN and leads to the proteasome-dependent degradation of CTTN.

### 3.6. CTTN is a Functional and Major Target of UCHL1 in NPC

**Figure 6:**
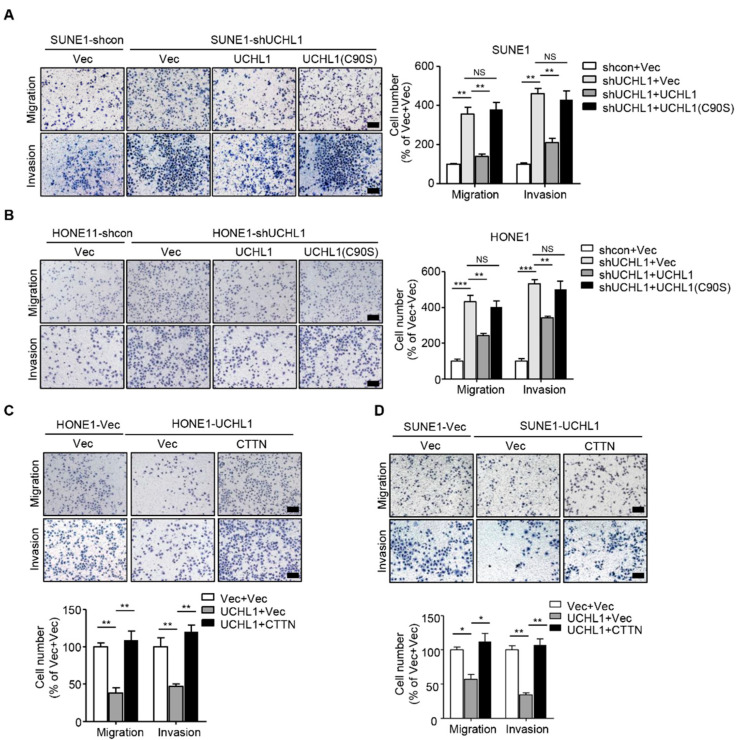
CTTN is a functional and major target of UCHL1 in NPC. (**A**,**B**) Transwell assay performed with SUNE1 (**A**) or HONE1 (**B**) cells stably transfected with control or shUCHL1 and reconstructed with vector, UCHL1, or UCHL1(C90S) in the presence (invasion) or absence (migration) of Matrigel. (**C**,**D**) Transwell assay performed with SUNE1 (**C**) or HONE1 (**D**) cells that stably transfected with vector or UCHL1 with reconstructed with vector or CTTN in the presence (invasion) or absence (migration) of Matrigel. Scale bar: 100 µm; Mean ± S.D.; * *p* < 0.05, ** *p* < 0.01, *** *p* < 0.001, NS: no significance compared with vector; Student’s *t*-tests. Data are representative of at least three independent experiments.

To examine whether the ligase activity of UCHL1 is involved in suppressing NPC cell migration and invasion, we performed transwell assays with wild-type UCHL1 and the enzymatically inactive mutant UCHL1 (C90S) in SUNE1 and HONE1 cells exhibiting stable UCHL1 knockdown. Transfection with the UCHL1(C90S) mutant failed to rescue the tumor-promoting effect of NPC cell migration and invasion (Figure 6A,B), which implied that the ligase activity of UCHL1 is indispensable for UCHL1 mediated-suppressive effects on NPC cell migration and invasion.

CTTN plays an important role in tumor progression in a number of malignancies, including esophageal carcinoma [24], hepatocellular carcinoma [13], and head and neck squamous cell carcinomas [25]. Whether CTTN promotes progression in NPC remains unknown. Because the degradation of CTTN is accelerated in UCHL1 over-expressing NPC cells, we hypothesized that supplementation of CTTN in SUNE1 and HONE1 that stably overexpress UCHL1 would restore the suppressive effects of UCHL1 on NPC cell migration and invasion. We performed transwell assays we found that the suppressive effects of UCHL1 on NPC cell migration and invasion were significantly rescued with reintroduction of CTTN into UCHL1 overexpressing NPC cells (Figure 6C,D). These findings demonstrate that CTTN is a functional and major target of UCHL1 in NPC cells.

### 3.7. UCHL1 Suppresses NPC Cell Invasion and Metastasis In Vivo

**Figure 7:**
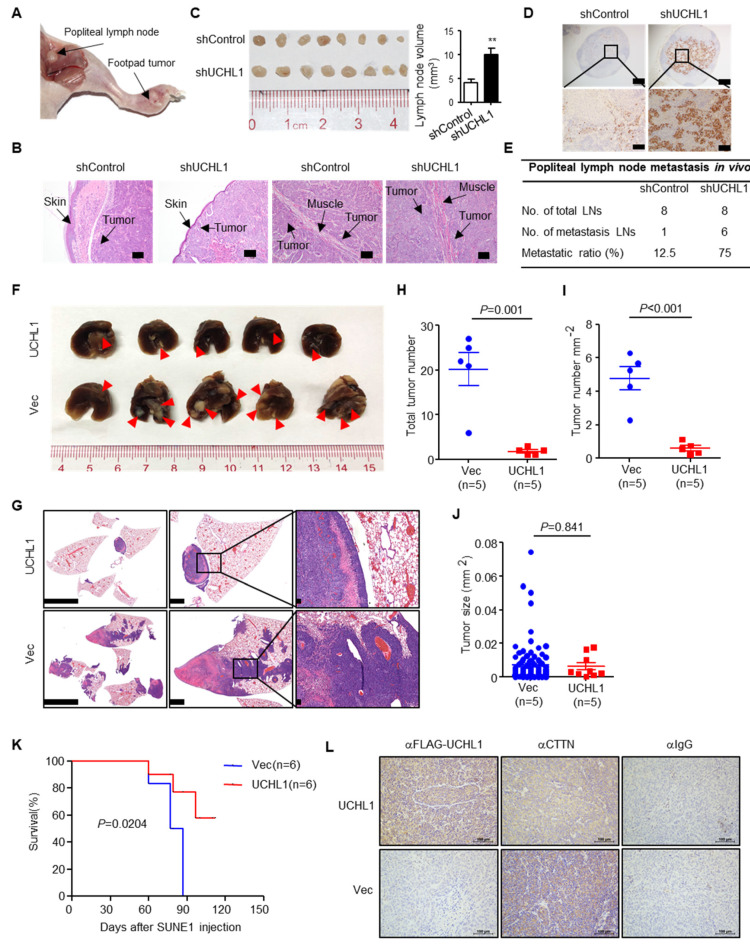
UCHL1 suppresses NPC metastasis in vivo. (**A**–**E**) Popliteal lymph node metastasis model was employed by injecting SUNE1 (3 × 10^5^) cells stably expressing shUCHL1 or control shRNA (shControl) into the footpads of nude mice (n = 8 per group). Representative image of primary foot-pad tumor and metastatic popliteal lymph nodes (A). H&E staining analysis of primary foot-pad tumor invaded in skin and muscle; Scale bar: 100 μm (B). Image and size of metastatic popliteal lymph nodes; Mean ± S.D.; Student’s *t*-tests. (C). Immunohistochemical staining and metastatic ratios of pan-cytokeratin-positive tumor cells in popliteal lymph nodes; Scale bar: 50 μm (D,E). (**F**–**J**) Lung metastasis (F), H&E staining (G), number (H), density (I), and size (J) of tumors metastasized in the lungs of nude mice at day 55 after intravenous injection with SUNE1 cells (1 × 10^6^) stably transfected with plasmids encoding vector or UCHL1 (n = 5 per group). (**K**) Survival analysis of nude mice intravenously injected with SUNE1 cells (1 × 10^6^) stably transfected with plasmids encoding (E) vector or UCHL1 (n = 6 per group). (**L**) Immunohistochemical staining for IgG, FLAG-UCHL1 and CTTN in lung metastases. Scale bar: 100 μm; Student’s *t*-tests. Each mouse sample was considered as an independent experiment; Three technological replicates were repeated in each sample.

To evaluate the effects of UCHL1 on NPC metastasis in vivo, we constructed a popliteal lymph node metastasis model by transplanting SUNE1 cells stably expressing shUCHL1 or control shRNA (shControl) into the footpads of nude mice (n = 8 per group). Six weeks later, the primary tumors and popliteal lymph nodes were obtained for analyzing (Figure 7A). H&E staining exhibited that the primary tumors in the UCHL1 knockdown group showed a more aggressive phenotype with invasion of the tumor cells towards the skin and muscle than the tumors in the shControl group (Figure 7B). Furthermore, the volumes of the popliteal lymph nodes were bigger, and the pan-cytokeratin-positive tumor cells were more in the UCHL1 knockdown group than in the shControl group (Figure 7C,D). Strikingly, the metastatic popliteal lymph nodes ratio was remarkably higher in the UCHL1 knockdown group than in the shControl group (Figure 7E).

To further evaluate the effects of UCHL1 on NPC metastasis in vivo, we employed lung metastasis colonization models. Mice injected with SUNE1 stably overexpressing UCHL1 exhibited significantly reduced metastatic tumor colonies in the lung compared to those injected with control cells (Figure 6F–I). The sizes of metastatic tumors were not affected by overexpression of UCHL1 (Figure 6J), although UCHL1 has been reported to inhibit NPC cell growth and induce apoptosis in vitro [7], indicating a primary role of UCHL1 in suppressing metastasis in vivo. Consistently, the mice injected with SUNE1 that stably overexpressed UCHL1 survived longer than those injected with control cells (Figure 6K). Hence, UCHL1 suppresses NPC cell metastasis both in vitro and in vivo.

To further examine whether UCHL1 suppresses CTTN expression in vivo, IHC was used to assess UCHL1 and CTTN protein levels in lung metastases. CTTN expression was significantly decreased in the UCHL1-overexpressed group compared with the vector group (Figure 7L). Taken together, these findings suggest that UCHL1 up-regulation is associated with a reduction in CTTN expression in vivo.

## 4. Discussion

In our study, we validate that UCHL1 is downregulation in both NPC cell lines and freshly frozen tissues as a results of its promoter hypermethylation. Overexpression of UCHL1 inhibited the migration and invasion of NPC cells, while knockdown of UCHL1 promoted NPC cell migration and invasion in vitro. Restoration of UCHL1 could suppress NPC metastasis in vivo, while CTTN functions as the downstream target of UCHL1 to regulate NPC metastasis. UCHL1 interacts with CTTN and targets CTTN for ubiquitination and promotes CTTN degradation to inhibit NPC metastasis. Our findings provide new insights into potential mechanism of UCHL1 regulating cell metastasis and clinical treatment for NPC.

Methylation modification plays an important role in NPC metastasis, but the mechanisms remain poorly understood. We have previously reported that hypermethylation of the HOPX, RAB37, and SHISA3 genes might serve as molecular biomarkers to predict NPC metastasis [17,26,27]. Therefore, the mechanisms and significance of methylation regulation in NPC are worthy to be study in greater detail.

UCHL1 has two enzyme activities and belongs to the DUB family involved in regulating the dynamic balance of ubiquitin in vivo [3]. Dysregulation of protein ubiquitination is associated with a wide range of human diseases, including cancer [28]. UCHL1 inhibits cell proliferation by deubiquitinating p53/HIF-a in hepatocellular carcinoma and by ubiquitinating MDM-2 in NPC [6,7]. However, there is limited knowledge regarding the role and mechanisms of UCHL1 in NPC metastasis. Based on previous microarray data, we found that UCHL1 is hypermethylated in NPC. We report significant UCHL1 downregulation in NPC tissues and cell lines, as indicated by qPCR and western blotting assay. Moreover, according to analysis from the TCGA dataset, UCHL1 is downregulated in a wide range of cancer types, including colon adenocarcinoma, kidney renal clear cell carcinoma, kidney renal papillary cell carcinoma, and bladder urothelial carcinoma (Supplementary Figure S3A). The MethHC database showed that the UCHL1 promoter is also hypermethylated in these cancers, and there are strong negative correlations between UCHL1 promoter methylation and mRNA expression in these cancers as well (Supplementary Figure S3B and S3C). Consistent with the findings in hepatocellular carcinoma [8] and prostate cancer [29], we hypothesized that UCHL1 functions as a tumor suppressor gene in NPC. Although Li et al. found that UCHL1 could inhibit NPC cell proliferation by promoting p53 signaling in vitro [7], the roles and mechanisms of UCHL1 in NPC in vivo remain undefined. Our study revealed that UCHL1 suppresses NPC metastasis in vitro and in vivo. We also demonstrate that UCHL1 hypermethylation contributes to its downregulation and leads to metastasis in NPC.

Based on mass spectrometry data, we confirmed that CTTN has the highest interaction score and physically interacts with UCHL1 in NPC. CTTN is an F-actin binding protein that promotes actin assembly [11]. CTTN has been involved in many cellular processes, including cell adhesion, cell proliferation, cell migration and invasion [30]. Therefore, dysregulation of CTTN causes many diseases, especially cancer. Mader et al. have been reported that EGF-induced CTTN phosphorylation promoted matrix proteolysis-dependent tumor cell invasion [31]. Chih-Chen et al. have reported that upregulation of CTTN expression by VEGF-C enhanced the invasive properties of esophageal squamous cell carcinoma in vitro and tumor metastasis in vivo [32]. As UCHL1 interacts with CTTN (Figure 4) and UCHL1 itself owns two opposite enzyme activities [4], we then asked if UCHL1 could regulate the stability of CTTN in NPC. We found that overexpression of UCHL1 could promote the degradation of CTTN (Figure 5C,D). Further ubiquitin assays showed that UCHL1 (WT) but not UCHL1 (C90S) mutant could increase the K48-linked ubiquitin level of CTTN, confirming that UCHL1 regulated the stability of CTTN through ubiquitin-proteasome pathway (Figure 5E–H). To explore whether CTTN is a functional target of UCHL1, we performed rescue assays in NPC cells and demonstrated that UCHL1 suppressed NPC metastasis by promoting the degradation of CTTN (Figure 6). As CTTN could promote actin assembly, further exploration could be done to see if UCHL1-CTTN interaction would affect actin assembly to promote cell movement and migration in NPC. Furthermore, Seema et al. have been reported that CTTN could control exosome secretion from cancer cells [33]. As exosome are small vesicular structures that contain a variety of components (including DNA, RNA, and proteins). Numerous studies have shown that these substances in exosomes are involved in growth, metastasis and angiogenesis in many cancer types [34]. Therefore, whether the UCHL1-CTTN interactions would affect exosome secretion in NPC remains unclear and may be an interesting project in our future study.

## 5. Conclusions

In summary, we identified a key oncogenic role of UCHL1 hypermethylation in NPC metastasis via the UCHL1/CTTN axis, which provides a novel therapeutic target for NPC treatment.

## Supplementary Materials

The following are available online at https://www.mdpi.com/2073-4409/9/3/559/s1, Figure S1: The UCHL1 is hypermethylated in nasopharyngeal carcinoma tissues. Figure S2: UCHL1 is hypermethylated in nasopharyngeal carcinoma cell lines. Figure S3: Promoter hypermethylation mediates downregulation of UCHL1 in four types of solid tumor. Figure S4: UCHL1 targets CTTN for K48-linked ubiquitination. Figure S5: Hypermethylation of UCHL1 suppresses degradation of CTTN. Table S1. Primers used in this study. Table S2. Sequencing of anti-FLAG-UCHL1 immunoprecipitation complex by mass spectrometry. The microarray data used in this study were downloaded from the GEO database using the GSE52068 and GSE62336 accession numbers. The UCHL1 expression data in other cancer types were obtained from The Cancer Genome Atlas (TCGA) database. The methylation level of UCHL1 and the correlation between expression and methylation of UCHL1 were analyzing by MethHC database. The authenticity of this article has been validated by uploading the key raw data onto the Research Data Deposit public platform (http://www.researchdata.org.cn), with the approval RDD number as RDDB2020000812.
