# Correction: Zhao et al. Hypermethylation of UCHL1 Promotes Metastasis of Nasopharyngeal Carcinoma by Suppressing Degradation of Cortactin (CTTN). *Cells* 2020, *9*, 559

**DOI:** 10.3390/cells13100816

**Published:** 2024-05-10

**Authors:** Yin Zhao, Yuan Lei, Shi-Wei He, Ying-Qin Li, Ya-Qin Wang, Xiao-Hong Hong, Ye-Lin Liang, Jun-Yan Li, Yang Chen, Wei-Jie Luo, Pan-Pan Zhang, Xiao-Jing Yang, Qing-Mei He, Jun Ma, Na Liu, Ling-Long Tang

**Affiliations:** Sun Yat-sen University Cancer Center, State Key Laboratory of Oncology in South China, Collaborative Innovation Center of Cancer Medicine, Guangdong Key Laboratory of Nasopharyngeal Carcinoma Diagnosis and Therapy, Guangzhou 510060, China; zhaoyin@sysucc.org.cn (Y.Z.); liangyl@sysucc.org.cn (Y.-L.L.); lijy1@sysucc.org.cn (J.-Y.L.); chenyang1@sysucc.org.cn (Y.C.); luowj1@sysucc.org.cn (W.-J.L.); zhangpp@sysucc.org.cn (P.-P.Z.); yangxiaoj@sysucc.org.cn (X.-J.Y.); heqm@sysucc.org.cn (Q.-M.H.); majun2@mail.sysu.edu.cn (J.M.); liun1@sysucc.org.cn (N.L.)

## Error in Figure

In the original publication [1], there were mistakes in Figures 3 and 6 as published. Incorrect images were used for the invasion-SUNE1-shcon group in Figure 3F, migration-SUNE1-shUCHL1+C90S group in Figure 6A, invasion-HONE1-Vec+Vec and UCHL1+CTTN groups in Figure 6C, and the migration-SUNE1-UCHL1+CTTN group in Figure 6D. These errors have been corrected. The corrected Figure 3 and Figure 6 appear below. The authors state that the scientific conclusions are unaffected. This correction was approved by the Academic Editor. The original publication has also been updated.

## Figures and Tables

**Figure 3:**
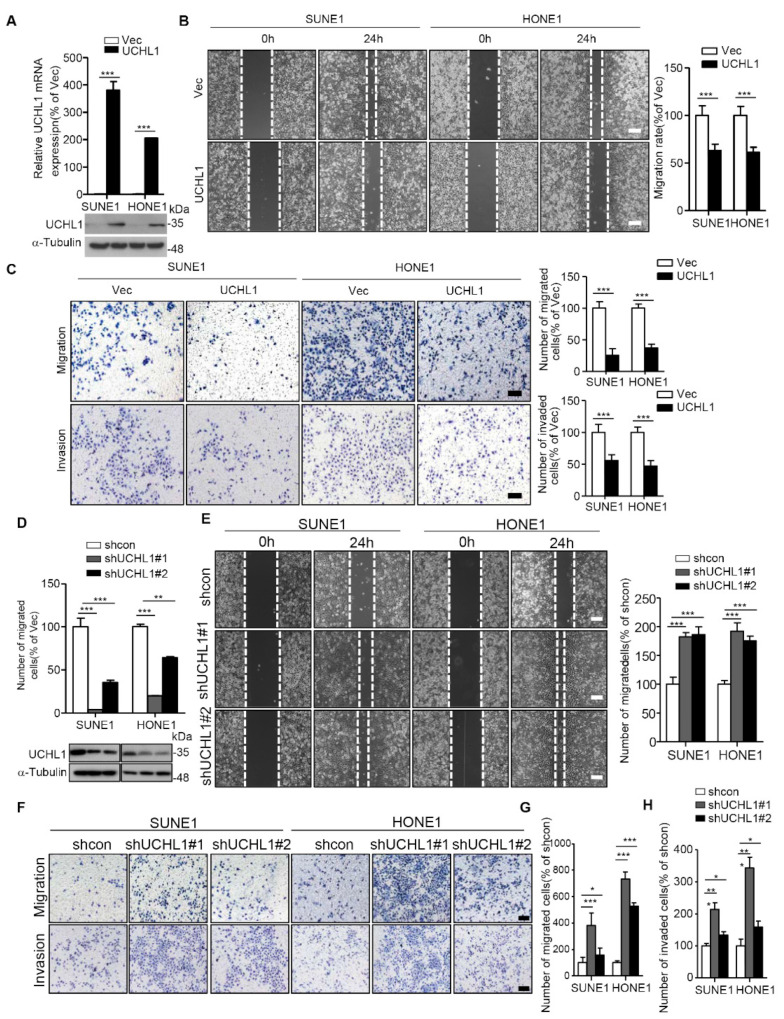
UCHL1 inhibits the migration and invasion of NPC cells in vitro. (**A**) qRT-PCR analysis of UCHL1 mRNA (up) and immunoblot analysis of UCHL1 and α-tubulin (down) in SUNE1 and HONE1 cells transfected with empty vector or plasmid encoding UCHL1. (**B**,**C**) Cell migration was measured using a wound healing assay (×200) (**B**) and transwell assay (×200) without Matrigel (**C**). Invasion was measured using a transwell assay with Matrigel (×200) (**C**). (**D**) qRT-PCR analysis of UCHL1 mRNA (up) and immunoblot analysis of UCHL1 and α-tubulin (down) in SUNE1 and HONE1 cells transfected with control or shUCHL1(#1 and #2) that stably overexpressed UCHL1. (**E**–**H**) Cell migration was measured using a wound healing assay (**E**) and transwell assay without Matrigel (**F**,**G**). Invasion was measured using a transwell assay with Matrigel (**F**,**H**). Scale bar:100 µm; Mean ± S.D.; * *p* < 0.05, ** *p* < 0.01; *** *p* < 0.001; Student’s *t*-tests. Data are representative of at least three independent experiments.

**Figure 6:**
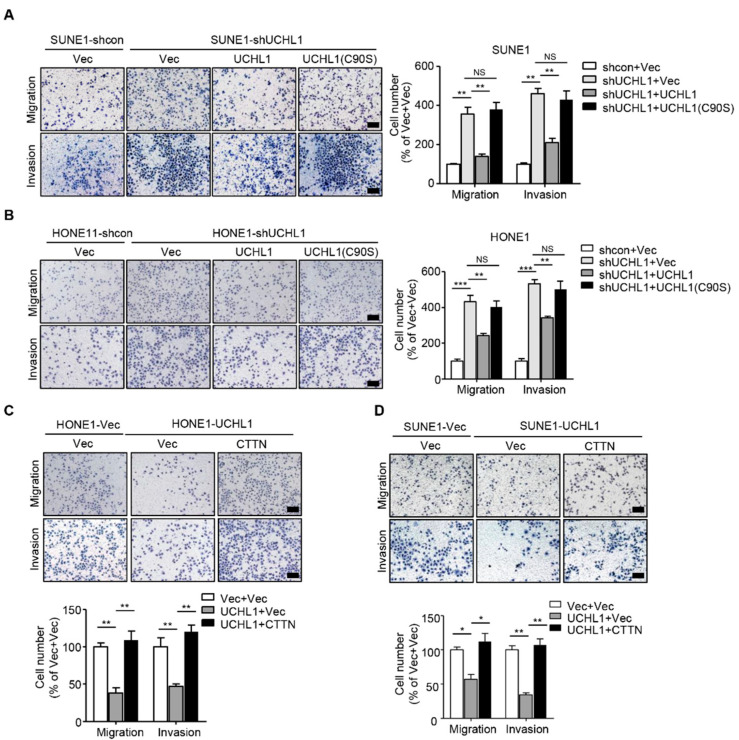
CTTN is a functional and major target of UCHL1 in NPC. (**A**,**B**) Transwell assay performed with SUNE1 (**A**) or HONE1 (**B**) cells stably transfected with control or shUCHL1 and reconstructed with vector, UCHL1, or UCHL1(C90S) in the presence (invasion) or absence (migration) of Matrigel. (**C**,**D**) Transwell assay performed with SUNE1 (**C**) or HONE1 (**D**) cells that stably transfected with vector or UCHL1 with reconstructed with vector or CTTN in the presence (invasion) or absence (migration) of Matrigel. Scale bar: 100 µm; Mean ± S.D.; * *p* < 0.05, ** *p* < 0.01, *** *p* < 0.001, NS: no significance compared with vector; Student’s *t*-tests. Data are representative of at least three independent experiments.
